# Characterization of the bacteriophages binding to human matrix molecules

**DOI:** 10.1016/j.ijbiomac.2017.12.052

**Published:** 2018-04-15

**Authors:** Chandni Porayath, Amrita Salim, Archana Palillam Veedu, Pradeesh Babu, Bipin Nair, Ajith Madhavan, Sanjay Pal

**Affiliations:** School of Biotechnology, Amrita Vishwa Vidyapeetham, Kollam, Kerala, 690525, India

**Keywords:** Gelatin, Heparin, Fibronectin, Bacteriophages, Human matrix

## Abstract

Recent literature has suggested a novel symbiotic relationship between bacteriophage and metazoan host that provides antimicrobial defense protecting mucosal surface by binding to host matrix mucin glycoproteins. Here, we isolated and studied different bacteriophages that specifically interact with human extracellular matrix molecules such as fibronectin, gelatin, heparin and demonstrated their potency for protection to host against microbial infections. We showed that subpopulations of bacteriophages that work against clinical isolates of *Escherichia coli* can bind to pure gelatin, fibronectin and heparin and reduced bacterial load in human colon cell line HT29. The bacteriophages were characterized with respect to their genome sizes, melting curve patterns and host tropism (cross-reactivity with different hosts). Since, the bacteriophages are non-toxic to the host and can effectively reduce bacterial load in HT29 cell line their therapeutic potency against bacterial infection could be explored.

## 1 Introduction

Emergence of multidrug resistant pathogens jeopardized efficacy of several antibiotics and forced to think about alternative treatment of microbial infections like phage therapy. The potential role of endogenous bacteriophages in controlling pathogenic infections have been widely studied in recent years. Mammalians may host bacteriophages to protect themselves against bacterial pathogens [[1], [2]]. Barr et al., [3] have shown that certain bacteriophages bind to mucin-1, a complex extracellular matrix (ECM) glycoprotein, and protects the mice from enteric infections. Apart from the antibacterial activity of bacteriophages, their contributions in health and diseases including colorectal cancer are also well established. Bacteriophages have shown to bind the membranes of normal blood cells and cancer cells [4]. An *in vitro* study by Krystyna et al., where the migration of melanoma cells on fibronectin was shown to be inhibited by T4 phage and its substrain HAP1 phage preparations. These interactions could possibly be utilized for antibacterial therapies in cancer patients [5]. Also, the studies on possible interactions between phages and immune cells especially the phagocytes of innate immunity have shown that they do not down regulate the phagocytosis [2]. Unlike the pathogenic viruses, bacteriophages do not to induce phagocyte degranulation, inflammation and ROS production that damage uninfected tissues, as they have a different action of mechanism on the phagocytotic cells [[6], [7], [8], [9], [10], [11], [12], [13], [14]]. Hence they colonize all niches of the body and could manipulate our immune system since they bypass the epithelial cell layers and disseminate throughout our bodies. But still specific interaction mechanisms between the bacteriophages with human cells, organs and immune system is largely unknown [15]. Here, we demonstrated that bacteriophages that selectively bind to the ECM can possibly protect host from bacterial infection (suggested change). Among many complex molecules in ECM, we chose to focus three well studied macromolecules: gelatin (denatured collagen), fibronectin and heparin. Gelatin is partially hydrolyzed form of collagen, a heterogeneous mixture of water-soluble proteins of high molecular masses and convenient to work with compared to collagen which is insoluble in aqueous buffer. Gelatin binds to fibronectin with high affinity and used for purification of fibronectin or removal of fibronectin from body fluid, especially plasma [16]. Fibronectin is a high-molecular weight (∼440 kDa) glycoprotein of the ECM that binds to membrane receptor integrins, critical for cellular attachment to matrix. Fibronectin binds to many other ECM components such as collagen, fibrin, heparin,; and plays an important role in cell adhesion, growth, migration, wound healing and embryonic development [[17], [18], [19]]. Heparins are highly sulfated glycosaminoglycan (3–30 kDa) that act as anticoagulant produced by basophils and mast cells. It is one of the main components of catheter lock solution. Heparin binds different signaling molecules, growth factors, pathogens and cell adhesion molecules like fibronectin. The structural similarity with heparan sulfate suggests that heparin may have a multifunctional role in cell communication [[20], [21], [22]]. All these macromolecules play important role in microbial infections [[23], [24], [25]]

Very often the bacterial pathogens target these ECM proteins by proteases and cause inflammation in affected tissues [26]. For example, N-terminal domain of fibronectin has been shown to bind to more than hundred different fibronectin binding proteins (FBP) of bacteria [19]. It has also been shown that Pap31, a bacteriophage encoded surface protein of *Bartonella henselae* [27] binds to heparin- binding FnIII 13–14 segment of fibronectin [28].

We selected the *E. coli* phages which bind these macromolecules so that they can protect the host actively against their target, *E. coli*. Moreover, they can also potentially protect from non-target pathogens by preventing those pathogens from binding to the ECM molecules and then tested their efficacy in protecting host cells from *E. coli* infections under *in vitro* condition. Potentially they can be used in phage therapy in particular developing novel wound healing applications [[29], [30]].

## 2 Methods and materials

### 2.1 Reagents

Growth medium for bacterial cultures (LB broth, LB agar, EMB agar, SS agar, TCBS agar, Deca strength phage broth, Mueller Hinton Agar) and Bovine Serum Albumin (BSA) were purchased from Himedia Laboratories. DMEM medium, agarose resins (Gelatin–agarose, Heparin agarose and Sepharose 4B), Resazurin and Triton X 100 were procured from Sigma Aldrich. Fetal Bovine Serum (FBS), Penicillin-Streptomycin mixture (PenStrep from Gibco), Amphotericin B and SYBR green master mix 2X DyNAmo Color Flash were obtained from Gibco, Thermo Fisher Scientific. All other salts and other reagents were procured from Sigma Aldrich.

### 2.2 Bacterial strains used

*E. coli* ET strain, a clinical isolate, gifted by Dr. Bhabatosh Das, THSTI, Faridabad, was used for the selection and propagation of bacteriophages. Clinical strains of *Shigella dysenteriae, Salmonella enterica, Vibrio cholerae*, *Pseudomonas aeruginosa, Klebsiella pneumoniae* and a multidrug resistant *E. coli* (*E. coli* MDR obtained from Dr. Anil Kumar V, Microbiology department, AIMS, Kochi, Kerala) were used to check the cross infectivity (tropism) of the isolated bacteriophages. Bacterial strains were maintained in Luria-Bertani (LB) agar plates at 37 °C. In liquid cultures bacterial strains were grown in LB broth with 120 rpm shaking.

### 2.3 Isolation and characterization of bacteriophages

#### 2.3.1 Isolation of bacteriophages from sewage

A sample of settled sewage water was filtered using 0.22 μm pore size syringe filter (Millipore Corp.), and filtrate was enriched by adding 2.5 ml of Deca strength phage broth into 22.5 ml of filtrate and inoculating 1 ml of overnight grown host culture (*E. coli* ET). The resulting preparation was incubated at 37 °C for 24 h, after which the culture was centrifuged to pellet down the bacteria and the supernatant was filter sterilized using 0.22 μm syringe filter. The supernatant was checked for the activity on the *E. coli* culture by spot assay where 10 μl of the supernatant was spotted onto the surface of the host bacterial lawn, left to dry and incubated overnight at 37 °C. After incubation, the plates were checked for zone of lysis indicating the presence of phages [31].

#### 2.3.2 Estimation of titer of the bacteriophages

The titer of the bacteriophages against *E. coli* ET from enriched sewage was determined by plaque assay using agar overlay method [32]. The filter sterilized phage lysates were diluted in SM buffer (100 mM NaCl, 8 mM MgSO_4_·7H_2_O, 50 mM Tris-Cl (pH 7.5) and 0.01% (w/v) gelatin solution) to 10^−8^ dilution and 100 μl each of the diluted phages and the host bacteria (overnight culture) were mixed with 4 ml of soft agar (0.7% agar) which were then overlaid onto the solidified base LB agar plates (2% agar) and incubated overnight at 37 °C [22].

Individual plaques formed after incubation were further picked and enriched separately against *E. coli* ET and their respective titers were determined.

#### 2.3.3 Test for tropism of bacteriophages to host bacteria

The isolated bacteriophages were checked for their host specificity (tropism) by spot assay [mentioned above, in 2.3.1] against six enteric pathogens namely *Shigella dysenteriae, Salmonella enterica, Vibrio cholerae*, *Pseudomonas aeruginosa, Klebsiella pneumoniae* and multidrug resistant *E. coli* (*E. coli* MDR).

#### 2.3.4 Isolation of genomic DNA from bacteriophages and analysis by melt curve

We first isolated gDNA of the bacteriophages using conventional buffered phenol-chloroform extraction method [33] and quality of DNA was checked by agarose gel electrophoresis. Further characterization was carried out with melt curve analysis, assuming that unique phage DNA will have unique melting curve and *T_m_* (the temperature at which 50% of DNA is denatured) [34].

We used a SYBR green master mix 2X DyNAmo Color Flash (Thermo Scientific) to conduct melt-curve analysis. Reactions were carried out on a Step one plus real-time machine (ABI) and results analysed using Stepone software. The 15 μl reactions consisted of 7.5 μl SYBR green (Thermo Scientific), 100 ng DNA, and water. The melting profile was done using an initial annealing of 55 °C for 15 s followed by temperature ramping at 0.1 °C per step with a 15 s hold time.

### 2.4 Antibiotic sensitivity for host bacterial strains using Kirby Bauer Method

Antibiotic sensitivity of *E. coli* ET and *E. coli* MDR against 12 antibiotics belonging to different classes namely aminoglycosides [Gentamicin–10 μg/disc, Streptomycin–10 μg/disc], 2nd generation Cephalosporin [Cefoxitin −30 μg/disc], 3rd generation cephalosporin [Ceftazidime–30 μg/disc], β-lactams [Penicillin–6 μg/disc, Amoxicillin/clavulanic acid–30 μg/disc (Amoxiclav), Ampicillin–10 μg/disc, Methicillin–10 μg/disc], glycopeptides [Vancomycin–30 μg/disc, fluoroquinolone [Ciprofloxacin–5 μg/disc], sulphonamides [Co-trimoxazole–25 μg/disc], tetracyclines [Tetracycline–30 μg/disc] were tested.

### 2.5 Binding of bacteriophages to fibronectin by plate binding assay

Fibronectin (Fn) was purified from human blood plasma (plasma samples were obtained from Regional Cancer Center, Kerala, India) using gelatin-agarose column chromatography [35]. Fibronectin binding phages were isolated using gelatin affinity column chromatography. Phage suspension was passed through Fn saturated gelatin agarose column and the excess lysate was removed using wash buffer. The phages were then eluted with 1 M NaCl (1 × 10 bed volumes) and then subsequent elution using 3% DMSO(1 × 3 bed volumes) [[36], [37]]. The eluted fractions were then checked for the number of plaque forming units by agar overlay method against the host culture. Individual plaques from the DMSO eluted fraction were then enriched and further studied for their Fn binding on 96 well plate. Fn (200 μg/ml) and BSA (200 μg/ml) each were coated on microtitre plate (100 μl each) and incubated for 5 h. After incubation the excess amount of proteins were decanted, washed twice with 200 μl PBS (phosphate buffered saline, made of 137 mM NaCl, 2.7 mM KCl, 10 mM Na_2_HPO4, 1.8 mM KH_2_PO4) and then added with phage lysate (titer 10^4^) and incubated for 2 h. The excess lysate was removed after incubation and then overlaid with equal volumes of *E. coli* culture and the absorbance at 600 nm was measured after 4 h of incubation.

### 2.6 Binding of bacteriophages to heparin and gelatin

Gelatin-agarose (≥3 mg/ml of agarose beads) and Heparin agarose beads (54-210 USP/ml of agarose beads) from Sigma Aldrich were used to study the binding of bacteriophages to gelatin and heparin. 100 μl of agarose beads and Heparin/Gelatin coated agarose beads were mixed with 200 μl of phage lysate (titer 10^6^) and incubated for 1 h under shaking condition at 37 °C. The resins were allowed to settle down and excess lysate was removed. The resins were washed three times with 1 ml PBS to remove the non-adherent phages. The resins were then resuspended in equal volumes of *E. coli* culture and the absorbance at 600 nm was measured at different time intervals.

### 2.7 *Ex vivo* antimicrobial activity assay

Human blood plasma was diluted three times and passed through gelatin agarose column to remove Fn and the flow through (Fn depleted plasma) was collected. Plasma and Fn depleted plasma (250 μl each) were mixed with 30 μl each of *E. coli* ET (OD_600_ 1) and its phage and kept for incubation at 37 °C along with Resazurin (10% of the total mixture). Fluorescence at 530/590 nm was taken at different time intervals to study the protection against host culture by bacteriophages in plasma.

### 2.8 Maintenance of mammalian cell line HT29

The human colon adenocarcinoma (HT29) cell lines obtained from NCCS, Pune, India, were cultured in complete DMEM medium supplemented with 10% Foetal Bovine Serum, 1% Penicillin Streptomycin mixture (PenStrep from Gibco) and 0.2% Amphotericin B (Gibco) in a humidified atmosphere containing 5% CO_2_ at 37 °C ^.^

### 2.9 Cytotoxicity of bacteriophages on mammalian cell lines (HT29 cells)

Bacteriophages against pathogenic *E. coli* (*E. coli* ET) were used to treat mammalian cell lines to check for their cell toxicity. HT29 cells (colorectal cancer cell line) were incubated in 96 well plate with a seeding density of 10^4^ cells per well at 37 °C and 5% CO_2_ for 24 h. The cells were then washed twice with growth media and 100 μl each of phage lysate against *E. coli* ET (10^4^ CFU/mL), heat inactivated phage lysate, PBS, Triton X 100 (final concentration of 1% in the media) and media were added into the wells and incubated at 37 °C and 5% CO_2_. Resazurin assays were done after 2 h and 24 h of treatment to check the viability of the cells.

### 2.10 Protection of HT29 cells by isolated bacteriophages against host pathogen

Isolated phages were also checked for their role in protection from infection against host pathogens in HT29 cell line. Cells were seeded onto 96 well plate with a seeding density of 10^4^ cells per well at 37 °C and 5% CO_2_ and after 24 h incubation the cells were rinsed with PBS to remove the residual antibiotics and layered with 100 μl of serum free media containing *E. coli* ET phages (10^4^ PFU/ml) for 24 h at 37 °C and 5% CO_2_. The cells were washed thrice with PBS to remove non adherent phages and both phage treated and untreated cells were further incubated with 100 μl serum-free media containing *E. coli* ET (OD_600_ 0.2) in 96 well plate at 37 °C and 5% CO_2_ with 0.2% Resazurin. After 3 h of incubation, fluorescence was taken at 530/590 nm to check for the reduction in bacterial growth in treated cell lines.

### 2.11 Statistical analysis

Graph Pad Prism was employed to perform the statistical analysis. The results of experiments were expressed in terms of mean values ± standard deviations.

## 3 Results and discussion

### 3.1 Characterization of bacteriophages based on their tropism, titer, genomic size and melt curve analysis of the bacteriophage DNA

**Fig. 1:**
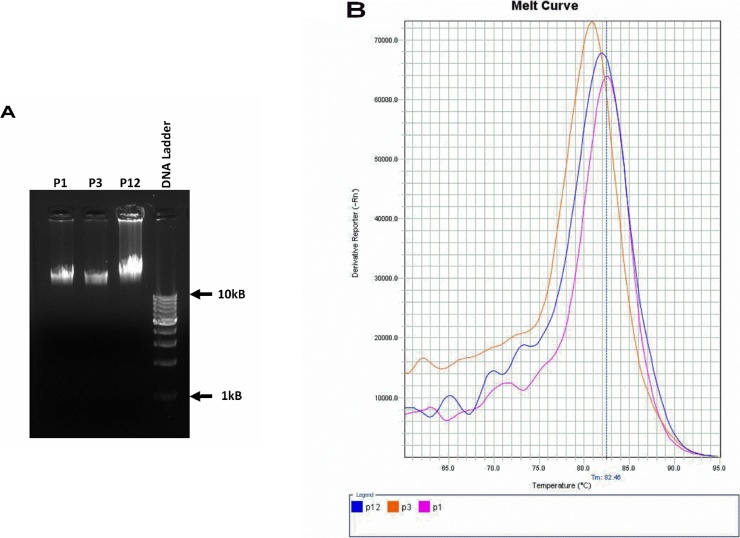
Characterization of the phage genome (A) Agarose gel electrophoresis of the nucleic acid isolated phages P1, P3 and P12 showing approximate genome size (B) Melt curve profile of the nucleic acids from the phages.

The enriched sewage lysates against *E. coli* ET were subjected to plaque assays, individual plaques were enriched and their titers were determined. The phages were then checked for their cross infectivity against six other enteric pathogenic bacteria (Table 1). Three phages (designated as P1, P2 and P12) isolated against *E.coli* ET were found to be cross reactive against *E. coli* MDR and were propagated for further experiments. P12, apart from *E. coli* MDR was found to be cross reactive with *Klebsiella pneumoniae* (suggested change). The phage titers against *E. coli* ET were found to be 3 × 10^6^ PFU/ml for phage P1 and P3 and 8 × 10^4^ PFU/ml for phage P12. Nucleic acid was isolated from the phages P1, P3, and P12; and were analysed for their approximate genome size. For further characterization, the phage genomes melt curve analysis were carried out. The Tm for the three phages, P1, P3, and P12 were 82.61 °C, 80.81 °C and 82.31 °C respectively (Fig. 1). Though Melt Curve analysis is a qualitative assay and does not identify the specific change in the sequences, it has a very short turnaround time and therefore a quicker method to characterize phages based on their genome [38].

**Table 1:** Cross infectivity of the isolated bacteriophages against *E. coli* ET (P1, P3 and P12) on other clinical pathogens. P1 and P3 showed cross reactivity to only *E. coli* MDR whereas P12 cross reacted with *Klebsiella pneumoniae* as well.

Bacterial Strains	Phages		
	P1	P3	P12
*E coli* ET	+	+	+
*E coli* MDR	+	+	+
*Vibrio cholerae*	−	−	−
*Salmonella enterica*	−	−	−
*Shigella dysenteriae*	−	−	−
*Klebsiella pneumoniae*	−	−	+
*Pseudomonas aeruginosa*	−	−	−

### 3.2 Antibiotic sensitivity profile of the host bacteria

Host strains (*E. coli* ET and *E. coli* MDR), were characterized by their antibiogram to determine the antimicrobial resistance profile. *E. coli* MDR was resistant to most of the antibiotics except aminoglycosides and tetracyclines compared to *E. coli* ET (resistant to class Penicillins) (Table 2). Antibiogram profile helps in choosing the antibiotic if combination therapy (phage and antibiotic) protocol needs to be developed. The polyvalent nature of the phages and their tropism towards MDR pathogenic bacteria makes them an ideal candidate to tackle antibiotic resistance widely reported in nosocomial infections [39].

**Table 2:** Antibiotic sensitivity profile (Antibiogram).

Bacterial strains	Antibiotics											
	GEN	S	P	CX	AMC	VA	CIP	CA	CO	TE	A	MET
*E coli* ET	+	+	−	+	+	+	+	+	+	+	−	−
*E coli* MDR	+	+	−	−	−	−	−	−	−	+	−	−

Gentamicin – GEN, Streptomycin – S, Penicillin – P, Cefoxitin – CX, Amoxyclav – AMC, Vancomycin – VA, Ciprofloxacin – CIP, Ceftazidime – CA, Co Trimoxazole – CO, Tetracycline – TE, Ampicillin – A, Methicillin – MET.

Symbols: Antibiotic sensitive (+) and antibiotic resistant (−).

### 3.3 Binding of bacteriophages to fibronectin

**Fig. 2:**
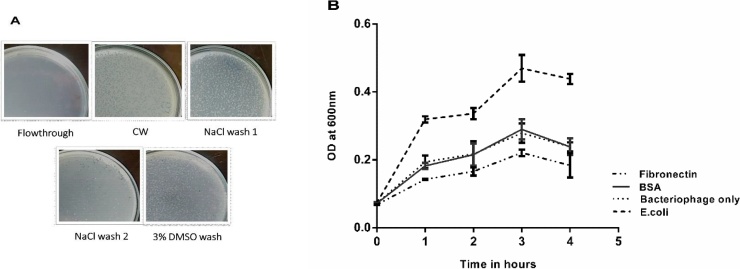
Binding of bacteriophages (against *E. coli* ET) to fibronectin. (A) Plaque forming units in the different fractions of elutions with wash buffer (CW), 1 M NaCl and 3% DMSO after column chromatography through Fn saturated gelatin agarose column. (B) Plate binding assay of bacteriophages to Fn where Fn and BSA (each with concentration 200 μg/ml) coated plates pre-treated with bacteriophages were overlaid with host pathogen and there was around 23% more reduction in their growth in Fn coated wells after 4 h of incubation.

To check if the isolated bacteriophages could bind to Fn, phage suspension was passed through Fn saturated gelatin agarose column and eluted with 1 M NaCl, subsequently eluted with 3% DMSO and the eluted fractions were subjected to plaque assay. Plaques isolated from DMSO eluted fractions were further enriched and the titres were determined. Equal amounts of phage lysate (titer of around 10^4^) were added into the fibronectin coated 96 well plates and incubated. After incubation, plates were washed with PBS and 100 μl of *E. coli* culture was added into the wells and incubated further. A reduction in bacterial growth in the Fn coated wells indicates the presence of more bacteriophages bound to the fibronectin and there was around 23% more reduction in bacterial count as compared to that of wells coated with BSA (Fig. 2).

### 3.4 Binding of bacteriophages to gelatin and heparin

**Fig. 3:**
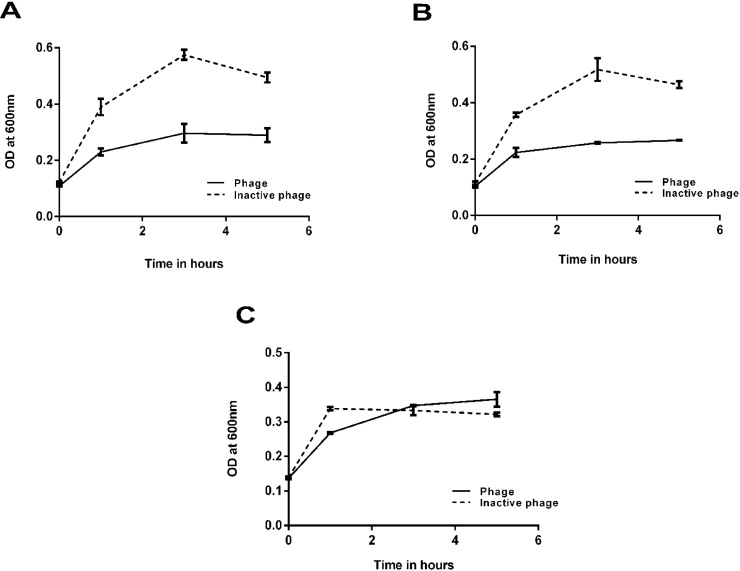
Binding of bacteriophages to heparin and gelatin. *E. coli* ET (host culture) treated with phage coated Heparin (A) and Gelatin (B) agarose beads showed around 50% reduction in the bacterial growth after 3 h of incubation at 37° C whereas agarose beads coated with phages (C) did not affect the growth of host culture showing the affinity of phages to heparin/gelatin.

Bacteriophage adherence to collagen/heparin was tested using agarose resins conjugated with gelatin/heparin. The host cells were incubated with equal volumes of resins (with or without phage treatment) to check for the reduction in the bacterial growth. Both the resins pre-treated with phages exhibited around 58% reduction in growth of the host bacterium after 5 h incubation at 37° C. To check if the phages adhered nonspecifically to the agarose part of the resin, the reduction in bacterial growth was checked with pure agarose (Sepharose 4B) beads which were pre-treated with or without phage along with the gelatin/heparin coated agarose beads. It was clearly observed that there was no significant reduction in the bacterial growth indicating the absence of nonspecific binding of phages to agarose (Fig. 3).

### 3.5 Ex vivo antimicrobial activity assay

**Fig. 4:**
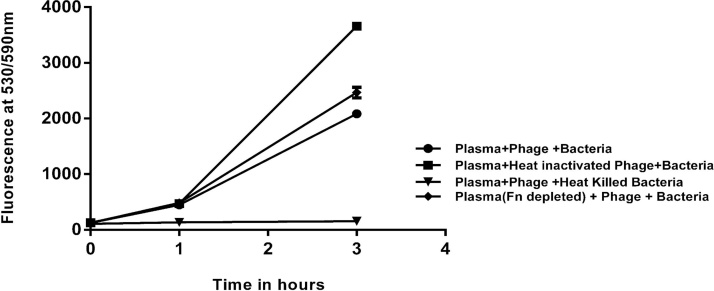
Infection by *E. coli* ET in plasma with specific phages was determined by Resazurin assay (fluorescence at 530/590 nm) for 3 h and compared to the control (heat inactivated phages), reduction in bacterial growth was observed by 43% in the phage treated plasma and 33% in the Fn depleted plasma.

The study was done to check the role of fibronectin in infection and to see if the presence of fibronectin can enhance the activity of phages to infect and reduce the growth of the host bacteria. Diluted human blood plasma (filter sterilized) was run through a gelatin agarose column to trap the gelatin binding proteins (major protein being fibronectin) and the flow through was collected. Both the diluted plasma and the Fn depleted plasma were then mixed with bacteriophages and the host bacterium and incubated for 3 h at 37° C. It was observed that there was 10% more reduction of bacterial growth in the diluted plasma (43%) than that of Fn depleted plasma (33%) when compared to the phage untreated plasma (Fig. 4).

### 3.6 Cytotoxicity of bacteriophages on mammalian cell lines (HT29 cells) and their protection against infection by host pathogen

**Fig. 5:**
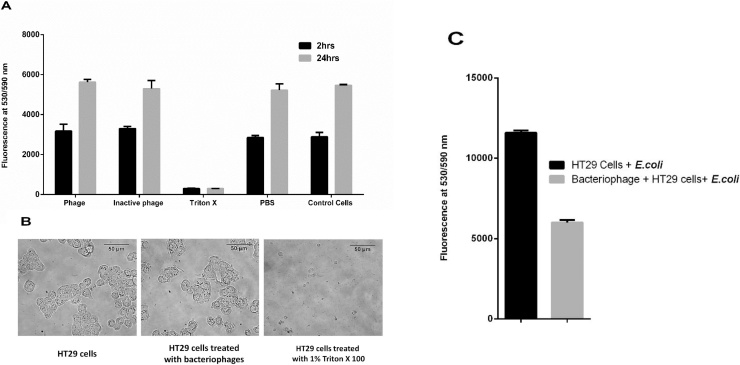
(A) Cytotoxicity of isolated bacteriophage (P1) on mammalian cells was studied with human colon adenocarcinoma (HT29) cells (10^4^ cells/well) using Resazurin assay (fluorescence at 530/590 nm). Graph shows the viability of mammalian cells with and without phage treatment. Treatment with phages did not affect the viability as compared to the positive control (1% Triton X 100). (B) Cell images show that there is no change in their morphology after phage treatment as compared to the controls. (C) Graph shows the reduction of bacterial pathogen in bacteriophage treated HT29 cell line (10^4^ cells/well) using Resazurin assay (fluorescence at 530/590 nm).

The isolated bacteriophages were checked for their toxicity on mammalian cell lines using HT29 cells. Cells were treated with phage lysate (P1), heat inactivated phage lysate, 1% Triton X (positive control) and PBS (negative control) where Resazurin assay showed similar results for phage treated as well as normal cells and exhibited similar cell morphology after 2 h and 24 h of incubation of the cells [Fig. 5 (A) and (B)]. They were then studied for their ability to protect mammalian cells against infection by pathogenic host on HT29 cells. The cells (seeding density of 10^4^ cells/well) were pre-treated with or without phages and checked for the reduction in the pathogenic host bacteria by Resazurin fluorometric assay and measuring the total fluorescence (of both mammalian and bacterial cells) at 530/590 nm. It was observed that there was around 48% reduction in total fluorescence in the phage pre-treated HT29 cells infected with host bacteria [Fig. 5(C)] indicating that the adhered phages could potentially reduce the bacterial load as compared to the phage untreated HT29 cells. Since the phages were shown to be non-toxic to the mammalian cells, the reduction in fluorescence should possibly be from the reduction in growth of bacteria and least intervention from loss of viability of mammalian cells.

Overall, we could successfully select a sub-population of bacteriophages which can bind to three major macromolecules of ECM which have crucial role in infection control in the vertebrate host. The phages can potentially be used to reduce infection in different microenvironment of the tissues. Many different challenges including regulatory issues remain to be sorted out before bacteriophage therapy is accepted in modern medicine, but our studies open up a new vista where we show that huge diversity of phages allow us to select bacteriophages based on their specific binding to host matrix macromolecules which can be used for those phage therapy. This will not only facilitate the control the target host bacteria against which phages have been obtained but also they can passively prevent the binding of other non-host bacteria which require binding to matrix molecules for their infection. For example, we know most of the bacteria studied bind to N-terminal domain of fibronectin [19]. We can select bacteriophages which can specifically bind to that specific N-terminal domain. As long as they occupy the concerned domain, infection by any bacteria via fibronectin can be prevented. But the blocking of these binding sites not necessarily improves the outcome of the health as those domains might involve in many other biological functions including the immunological clearance of the bacterial infection. Hence such use of phages need to be judged case by case basis. Nevertheless, it opens a vista of designing another class of biological ligands which so far has not been explored.

## 4 Conclusion

In this study, we have shown that certain subpopulation of bacteriophages isolated from sewage can bind to pure gelatin, fibronectin and heparin and reduced the *E. coli* load in the presence of these extracellular molecules. The phages were proved to be non-toxic and also they could protect the mammalian cells against the host pathogen infection. These matrix binding bacteriophages may, therefore, can potentially be considered for phage therapy against *Escherichia* infections in host. This is first report of its kind where bacteriophages were selected for its binding to host matrix macromolecules and opens up new ways of selecting bacteriophages for phage therapy.
